# Epidemiological Survey of Sinonasal Malignancy in North-East Iran

**Published:** 2015-05

**Authors:** Mehdi Poursadegh, Farid Poursadegh, Majid Esmaeili, Mehdi Bakhshaee

**Affiliations:** 1 *Department of Otorhinolaryngology- Head & Neck Surgery, Imam Reza Hospital, Faculty of Medicine, Mashhad University of Medical Sciences, Mashhad, Iran.*; 2 *Student of Medical Sciences, Imam Reza Hospital, Faculty of Medicine, Mashhad University of Medical Sciences, Mashhad, Iran.*; 3 *Sinus and Surgical Endoscopic Research Center, Ghaem Hospital, Faculty of Medicine, Mashhad University of Medical Sciences, Mashhad, Iran.*

**Keywords:** Adenoid Cystic, Carcinoma, Chemotherapy, Nose, Paranasal Sinuses, Squamous Cell, Surgery, Radiotherapy

## Abstract

**Introduction:**

Sinonasal malignancies are uncommon neoplasms with several histological subtypes. These malignancies have a poor prognosis and, because of the nonspecific nature of the symptoms, most patients are diagnosed late when the disease is already at an advanced stage. Therefore, most sinonasal malignancies tend to be treated with surgery and postoperative radiotherapy. Understanding the incidence and prevalence of clinical symptoms, pathology, diagnosis, and subsequent prognosis of the disease is important for early diagnosis.

**Materials and Methods:**

Medical records of patients with a confirmed diagnosis of sinonasal malignancy in a tertiary referral center from 1998 to 2009 were retrospectively investigated by chronological examination. Information relating to symptoms, pathology, and treatment of patients were collected from the checklists and used to generate tables and graphs, while descriptive statistical tests were used to compare data.

**Results:**

The records of 69 patients were examined, including 45 (65.2%) male and 24 (34.8%) female patients with a combined mean age of 54.07±16.04 years. Twenty-one patients (30.4%) were aged less than 45 years and 48 (69.6%) were more than 45 years of age. The most common symptom was facial swelling in 46 (66.6%) patients and the most common kind of tumor was squamous cell carcinoma in 28 (40.6%) patients. The primary location of the tumor in most patients was the maxillary sinus (54 patients; 78.3%). A majority of patients present in advanced stage (stage III or more) with intraorbital (39.1%) or intracranial (4.3%) involvement, or regional lymphatic (28.99%) or distance metastasis (7.2%). The most common treatment was surgery (17 patients; 24.6%).

**Conclusion:**

Due to their nonspecific symptoms, most sinonasal malignancies are diagnosed at an advanced stage of the disease. Therefore, all patients with nonspecific symptoms, especially older males, should be evaluated for sinonasal malignancies in order to eliminate this diagnosis.

## Introduction

With a worldwide annual incident rate of 1/200,000, sinonasal tract malignancy is a relatively rare disease (1). However, the incidence of these tumors may be greater in some parts of world, including Asia and Africa and especially Japan (2). In France, cancers of the nasal cavities and paranasal sinuses account for 2–3% of upper aerodigestive-tract cancers (3). Head and neck tumors are the sixth most common body cancer, and sinonasal tumors rank third in this area. In particular, malignant neoplasms of the nasal cavity and paranasal sinuses are the most common benign tumors of this region. Many risk factors are associated with this pathology, including exposure to chemical agents wood and leather and being involved with furniture industry (2,4-5).

These tumors have a poor prognosis, and mortality and relapse are common even after treatment due to common delays in diagnosis until the advanced stages of the disease (4, 6-10).

This is due, at least in part, to the nonspecific signs and symptoms of the malignancy, such as bleeding from the nose, blocked nose, and headache; symptoms which are commonly seen in benign pathologies in this area. The nonspecific and common clinical symptoms of head and neck tumors are often responsible for the long duration of, often inappropriate, treatment. A lack of knowledge of the signs and symptoms of such tumors among doctors combined with disregard of the early, nonspecific symptoms are the main reasons for the typical late diagnosis of the disease (8). 

Recent developments in methods and tools for the diagnosis and, in particular, prevention of this disease in the early stages, in addition to the development of improved endoscopy procedures, has allowed the possibility of the simple and accurate early detection of this disease. However, these improvements require the primary care physician to play a more active role in both understanding the early characteristics of this disease and ensuring early referral to specialists. Regional epidemiological studies on the incidence and risk factors are important in order to help primary care physicians refer a suspected patient to a specialist in the early stages of the disease, and thus improve quality of life and survival rates from these cancers.

## Materials and Methods

This descriptive retrospective study was performed by case-report method and by evaluation of all treated patients presenting with sinonasal malignant tumors from 1998 to 2009 at three referral centers of this disease in our study area from Information from patient records was made available to us, including age, gender, presentation, symptoms, pathology, location of involvement, risk factors, place of residence, eye and brain involvements, lymphatic node metastasis, and type of therapy. 

Data were analyzed using SPSS v13, and were presented by descriptive statistics into tables and figures. A Chi-square and independent T-test were used for comparison of variables. P<0.05 was considered statistically significant.

## Results

In the present study, we evaluated 69 patients, including 45 (65.2%) male and 24 (34.7%) female patients, with an overall mean age of 51.07±16.40 years (range, 17–83 years). In total, 51 patients (73.1%) lived in cities while 18 (26.9%) lived in rural areas. 

Drug addiction was the most common risk factor among patients, with 15 cases (21.70%), followed by smoking with eight cases (11.6%) and alcohol abuse with one case (1.3%). The most common clinical symptom observed was a swelling of the face in 46 cases (Table. 1). 

**Table 1:** The most common signs and symptoms of sinonasal malignancies among patients

**Clinical Symptoms**	**Frequency**	
	**Number**	**Percent**
Facial Swelling	46	66.6%
Nasal Obstruction	19	27.5
Headache	15	21.7
Bloody Rhinorrhea	13	18.8
Facial Pain	13	18.8
Neck Mass	13	18.8
Epiphorea	11	15.9
Rhinorrhea	11	15.9
Palatal Swelling	11	15.9
Proptosis	11	15.9
Sinus Tenderness	10	14.4
Facial Paresthesia	9	13.04
Nasal mass	9	13.04
Trismus	5	7.2
Palatal Ulcer	4	5.7
Dental luxation	4	5.7
Dental Pain	4	5.7
Anosmia	2	2.8
Diplopia	2	2.8
Chronic Otitis Media	1	1.4
Pharyngeal Mass	1	1.4
		

Among cranial nerves, trigeminal nerve was the most commonly affected with 20 cases (29%), followed by optic nerve in 11 cases (15.9%), facial nerve and abdocent were involved in three cases (4.3%). Among the 69 patients studied, squamous cell carcinoma in 28 patients (40.6%) was the most common tumor (Table. 2). 

The maxillary sinus tumor was the most common primary location in 54 patients (78.3%), followed by the ethmoid sinus in eight patients (11.6%), and nose in six patients (8.7%). There was no primary involvement of the sphenoid sinus or frontal sinus among our patients and one patient (1.3%) had a tumor of an unknown origin. 

At the time of referral, 27 cases (39.1%) presented with orbit involvement, three (4.3%) with intra-cranial involvement, and five (7.2%) with distant metastasis. Regarding treatment, surgery had been performed in 17 cases (24.6%), a combination of surgery and chemotherapy in 16 cases (23.1%), radiotherapy plus surgery in 15 cases (21.7%), chemotherapy and radiotherapy in 10 cases (14.4%), chemotherapy and surgery alone in four cases (5.7%).

**Table 2:** The sinonasal malignancies distribution according to histology

**Histologic Type**	**Frequency**	
	**Number**	**Percent**
Squamous Cell Carcinoma	28	40.6
Adenoid Cystic Carcinoma	8	11.6
Malignant Melanoma	6	8.7
Hodgkin Lymphoma	6	8.7
Osteosarcoma Chondroblastic	5	7.3
Undifferentiated carcinoma	4	5.8
Adenocarcinoma	3	4.3
Fibrosarcoma	2	2.9
Esthesioneuroblastoma	2	2.9
Malignant mixed tumor	1	1.4
Mucoepidermoid carcinoma	1	1.4
High grade sarcoma	1	1.4
Chondrosarcoma	1	1.4
Rhabdomyosarcoma	1	1.4

## Discussion

Sinonasal malignances usually occur with nonspecific symptoms, presenting not only a difficult diagnostic dilemma but also a therapeutic challenge. Over the course of 10 years of study, we investigated 69 patients with sinonasal malignant tumors presenting at our hospitals. As referral to other hospitals in the area is uncommon for these malignancies, it appears that these types of tumor are uncommon in the general population. Previous studies confirmed this inference, as Khademi et al identified 71 patients between 2000–2008 in Shiraz (11), Iran, while in Milwaukie, USA, Poetker et al found 40 cases between 1993–2003 (12).

We observed a male-to-female ratio of >1.8 (45 male vs. 24 female cases), with a mean age of 51.07±16.4 years old across both genders. Similar results have been reported in previous studies. For example, between 2001–2008 in Tehran, Motamed et al identified patients with a median age of 55 years (13), with a male frequency 1.6 times greater than that among female patients. Furthermore, in a study by Poetker et al (12), patients had a mean age of 53.2 years, with a male frequency 1.8 times greater than that among females. 

In our study, the clinical symptom which most commonly caused referral was a swelling of the face in 46 (63.8%) of patients. This was in contrast to the study of Motamed et al who found that obstruction of the nose was the first clinical symptom (13), while swelling of the face was the third most common clinical symptom in their study. In addition, in 2006 Fasunla et al found in Nigeria that epistaxis was the most common symptom (14), while swelling of the face was the fourth most common clinical symptom. This could be due to the neglect of common and nonspecific symptoms of these malignancies, causing detection of these tumors in advanced stages. 

In our study, maxillary sinus was the most commonly involved sinus (54 patients,78.3%), followed by the ethmoid sinus and nose without sphenoid and frontal involvement, respectively. Motamed et al found that the maxillary sinus was the most commonly involved sinus (13), followed by involvement of the sphenoid, ethmoid, and frontal sinus, respectively. Furthermore, as in our study, there are reports stating that the most common sinus involved in these malignancies are the maxillary and ethmoid sinus (13,15-20).

In the present study, we deduced that the most common malignant tumor with an epithelial origin was squamous cell carcinoma, with adenoid cystic carcinoma and adenocarcinoma following. In contrast, in previous studies by Motamed et al, Fasunla et al, and Ernest et al, adenocarcinoma was reported as the third most common tumor with an epithelial origin (13-15). 

Among non-epithelial tumors, Hodgkin’s lymphoma and malignant melanoma were the most frequent non-epithelial tumors. Zylka et al and Khandesi reported similar results, while Ernest et al reported lymphoma with a lower frequency (11,15,21). In the present study, 27 patients (39.1%) with eye involvement, three (4.3%) with intra-cranial and five (7.2%) with distant metastasis were reported. 

## Conclusion

Sinonasal malignant tumors have nonspecific symptoms in the nose and paranasal sinus, including nose obstruction and epistaxis, meaning that patients tend to be referred late to specialist treatment centers. Furthermore, the greatest frequency of sinonasal malignant tumors occurs in males with a median age of 51 years. Therefore, it is important that physicians carefully evaluate older symptomatic patients with sinonasal involvement, particularly men in the sixth decade of life.

## References

[1] Schröck A, Göke F, Van Bremen T, Kirsten R, Jakob M, Ehrenberg T (2012). Sinonasal tract malignancies: a 14-year single institution experience. HNO.

[2] Muir CS, Nectoux J (1980). Descriptive epidemiology of malignant neoplasms of nose, nasal cavities, middle ear and accessory sinuses. Clinical Otolaryngology & Allied Sciences.

[3] Schwaab G, Julieron M, Janot F (1997). Epidemiology of cancers of the nasal cavities and paranasal sinuses. Neurochirurgie.

[4] Comba P, Belli S (1992). Etiological epidemiology of tumors of the nasal cavities and the paranasal sinuses. Ann Ist Super Sanita.

[5] Harvey RJ1, Dalgorf DM (2013). Chapter 10: Sinonasal malignancies. Am J Rhinol Allergy.

[6] Kim CH, Song KS, Kim KS, Kim JY, Lee BJ, Lee JG (2005). Sulindac sulfide-induced apoptosis in sinonasal cancer cells. Acta Otolaryngologica.

[7] Zyłka S, Bień S, Kamiński B, Postuła S, Ziołkowska M (2008). Epidemiology and clinical characteristics of the sinonasal malignancies. Otolaryngol Pol.

[8] Betlejewski S, Bilewicz R, Stankiewicz C, Skorek A, Gierek T, Wardas P (2006). Malignant tumors of the nose and paranasal sinuses in the years 1992–2001. Otolaryngol Pol.

[9] Wang X, Shi G, Liu Y, Ji H, He M, Li J (2011). Analysis of the clinical and pathological characteristics of sinonasal neoplasms. Journal of Clinical Otorhinolaryngology, Head, and Neck Surgery.

[10] Jurkiewicz D, Wojdas A, Hermanowski M (2007). Malignant tumors of the nose and paranasal sinuses in the years 1971–2005 in the material of the Otolaryngology Clinic WIM. Otolaryngol Pol.

[11] Khandesi B, Moradi A, Hoseini S, Mohammadian panah M (2009). Malignant neoplasms of the sinonasal tract: Report of 71 patients and literature review and analysis. Oral Maxillofac Surg.

[12] Poetker DM, Toohil RJ, Loehrl TA, Smith TL (2005). Endoscopic management of sinonasal tumors: a preliminary report. Am J Rhinol.

[13] Motamed O (2009). Evaluation the frequency and clinical symptoms and surgeries of malignant paranasal tumors in hospitalized patients related to Tehran University of Medical Sciences between 2000 to 2009.

[14] Fasunla AJ, Lasisi AO (2007). Sinonasal malignancies: a 10-year review in a tertiary health institution. J Natl Med Assoc.

[15] Ernest A, Weymuller JR, Greg ED, Flint PW, Haughey BH, Lund VJ, Niparko JK, Richardson MA, Robbins KT (2010). Malignancies of the paranasal sinuses. Cummings Otolaryngology Head and Neck surgery.

[16] Hwang PH, Abdolkhani A, Snow JB, Wackym PA (2009). Embryology, anatomy and physiology of the nose and paranasal sinuses. Ballenger’s Otorhinolaryngology Head and Neck Surgery.

[17] Gerhard G, Rudolf P, Gerhard G, Heinrich I (2008). Anatomy, physiology, and immunology of the nose, paranasal sinuses and face. Basic otorhinolaryngology a step by step learning guide.

[18] Junqueira LC, Carneiro J, Junqueira LC, Carneiro J (2010). The respiratory system. Basic histology.

[19] Mandpe HA, Lalwani KA (2008). Paranasal sinus neoplasms. Otolaryngology Head and Neck Surgery Current Diagnosis and Treatment.

[20] Chalastras T, Elefteriadou A, Giotakis J, Soulandikas K, Korres S, Ferekidis E (2007). Non-Hodgkin’s lymphoma of nasal cavity and paranasal sinuses A clinicopathological and immuno- histochemical study. Acta Otorhinolarhingol Ital.

[21] Zylka S, Bein S, Kaminski B, Postula S, Ziolkowska M (2008). Epidemiology and clinical characteristics of the Sinonasal malignancies. Otolaryngol Pol.

